# Label-Free Protein Detection by Micro-Acoustic Biosensor Coupled with Electrical Field Sorting. Theoretical Study in Urine Models

**DOI:** 10.3390/s21072555

**Published:** 2021-04-06

**Authors:** Nikolay Mukhin, Georgii Konoplev, Aleksandr Oseev, Marc-Peter Schmidt, Oksana Stepanova, Andrey Kozyrev, Alexander Dmitriev, Soeren Hirsch

**Affiliations:** 1 Institute for Micro and Sensor Systems, Otto-von-Guericke-University Magdeburg, 39106 Magdeburg, Germany; 2 Department of Engineering, University of Applied Sciences Brandenburg, 14770 Brandenburg an der Havel, Germany; marc-peter.schmidt@th-brandenburg.de (M.-P.S.); soeren.hirsch@th-brandenburg.de (S.H.); 3 Department of Photonics, Saint Petersburg Electrotechnical University “LETI”, 197376 Saint Petersburg, Russia; gakonoplev@mail.ru (G.K.); oksana_lopatenko@mail.ru (O.S.); 4 FEMTO-ST Institute, CNRS UMR-6174, University Bourgogne Franche-Comté, 25000 Besançon, France; aleksandr.oseev@femto-st.fr; 5 Department of Physical Electronics and Technology, Saint Petersburg Electrotechnical University “LETI”, 197376 Saint Petersburg, Russia; mlpeltech@gmail.com; 6 Department of Ecological Physiology, Federal State Budgetary Scientific Institution “Institute of Experimental Medicine” (FSBSI “IEM”), 197376 Saint Petersburg, Russia; admitriev10@yandex.ru

**Keywords:** acoustic liquid sensor, shear bulk acoustic resonator, biosensor, structured sensor interface, electrical field manipulation, urine proteins characterisation

## Abstract

Diagnostic devices for point-of-care (POC) urine analysis (urinalysis) based on microfluidic technology have been actively developing for several decades as an alternative to laboratory based biochemical assays. Urine proteins (albumin, immunoglobulins, uromodulin, haemoglobin etc.) are important biomarkers of various pathological conditions and should be selectively detected by urinalysis sensors. The challenge is a determination of different oligomeric forms of the same protein, e.g., uromodulin, which have similar bio-chemical affinity but different physical properties. For the selective detection of different types of proteins, we propose to use a shear bulk acoustic resonator sensor with an additional electrode on the upper part of the bioliquid-filled channel for protein electric field manipulation. It causes modulation of the protein concentration over time in the near-surface region of the acoustic sensor, that allows to distinguish proteins based on their differences in diffusion coefficients (or sizes) and zeta-potentials. Moreover, in order to improve the sensitivity to density, we propose to use structured sensor interface. A numerical study of this approach for the detection of proteins was carried out using the example of albumin, immunoglobulin, and oligomeric forms of uromodulin in model urine solutions. In this contribution we prove the proposed concept with numerical studies for the detection of albumin, immunoglobulin, and oligomeric forms of uromodulin in urine models.

## 1. Introduction

Point-of-care (POC) analytical devices have been actively developing since 1990s and are widely applied in clinical practice. They provide rapid assessment, high portability, and easy to implement diagnostics ability to be used at a patient side. POC platforms can be based either on more conventional approaches as dipsticks or lateral-flow immunoassay or more sophisticated lab-on-chip and microfluidics technologies [1]. 

Urinalysis (urine tests) is one of the most convenient and affordable methods for routine diagnosis and screening of a broad spectrum of diseases since biological samples can be taken noninvasively and unrestrictedly, in most cases by a patient himself [2]. Above all, urinary biomarkers provide an essential tool for the diagnosis of nephrological and urological disorders. 

In general terms urine mainly consists of low molecular weight waste metabolic products, electrolytes and water produced from circulating blood in the processes of glomerular filtration and tubular reabsorption in the kidneys. Proteins and other large molecules could also be partially filtered but in physiological conditions more than 99% are reabsorbed: Normal urine contains very small amounts of proteins—less than 100 mg/L [3]. 

Urine proteomics has always been a subject of sufficient interest both by biochemists and medical researchers. Very sensitive methods such as two-dimensional gel electrophoresis [4], matrix-assisted laser desorption/ionization–time of flight mass spectrometry (MALDI-TOF MS) [5], liquid chromatography–mass spectrometry (LC–MS), tandem mass spectrometry (MS/MS) [6], high performance liquid chromatography (HPLC) [7], dynamic light scattering (DLS) [8,9,10], mass-spectrometry [11], electrophoresis [12], and enzyme-linked immunosorbent assay (ELISA) [13] were used for identification and quantification of urine proteins. About 6000 different proteins were identified in urine (and the concentration of more than 2500 proteins were estimated); some of them are clinically significant biomarkers of pathological conditions [14], although only very limited number of urinary proteins are determined in routine clinical practice.

With physiological proteinuria about two-thirds of the urine proteins comes from serum, mostly albumin (more than a half of all plasma proteins in urine), some immune globulins, and smaller molecular weight (<40 kDa) proteins, including microglobulins, hemoglobin and myoglobin. The remaining one-third, predominantly uromodulin, comes from the urogenital tract [15,16].

The most widely-diagnosed condition related to urinary proteins is albuminuria—excessive amounts of albumin in urine (more than 30 mg/L) which is associated with chronic kidney disease, diabetes, acute kidney damage, etc. [17,18,19]. Multiple methods and devices, from dipsticks to sophisticated microfluidic platforms based on chip electrophoresis, colorimetric optical detection, immunoassays and other techniques have been developed for the POC testing of microalbuminuria (urine concentration 30–300 mg/L) and macroalbuminuria (concentration more than 300 mg/L) [18,19,20,21,22]. 

Some of the conventional biochemical methods for urine analysis are sensitive to all plasma proteins, not only albumin and in routine diagnostics the term albuminuria is often used synonymously for proteinuria, which is not always correct because eleveted levels of other proteins, often with minor concentrations, have separate clinical significance. Various sensors for detecting such protein biomarkers in urine are also actively developing [23,24]. 

When proteinuria is non-selective (not only albumin, but other proteins are abnormally excreted) the urinary concentration of immunogloblins can also be suffiently increased, e.g., for some patients with massive proteinuria the concentration of immunogloblin G (IgG) can be as high as 1.5 g/L [25], while the typical range of IgG urinary concentrations is 0.25–5 g/L for subjects with norm- and mircoalbuminuria [26]. According to some researchers the ratio of albumin and globulin concentrations is a relevant diagnostic and prognostic factor [25,26,27].

Uromodulin is the most abundant protein in the urine of healthy individuals comprising about 40% of all protein compounds [28]. It was discovered in 1950 Igor Tamm and Frank Horsfall; during several decades it was extensively investigated but until recently its physiological role remained generally unclear [29]. Unlike many other urinary proteins like albumin or globulins uromodulin is not filtered from blood but produced by renal epithelial cells in the kidney itself, an estimated excretion range is 50–150 mg/day which corresponds to the urinary concentration 30–100 mg/L [28]. The molecular mass of the polypeptide is 85 kDa but in certain conditions it tends to form aggregates (oligomers) with much higher masses; it was shown that in native urine two oligomeric forms with molecular weights 7 MDa and 28 MDa are dominant [8].

For a long time uromodulin had been rather overlooked by medical professinals but recent researches showed that the levels of uromodulin in plasma and urine are valuable biomarkers of kidney and urinary tract diceases including chronic kidney dicease, urolithiasis, and some genetic disorders [30,31,32,33,34,35,36]. Various analytical techniques were used for detecting, qualitative determination and characterization of this protein in urine [7,8,9,10,11,12,13]. They all require costly equipment, disposables, reagents, highly qualified personal and involve comprehensive sample preparation procedures; only ELISA was introduced to clinical practice. Though recently microchip-based platforms based on ELISA were created for several protein biomarkers [37], no such devices have been reported yet for the uromodulin analysis in urine or plasma. 

Development and implementation of qualitative urine tests for uromodulin is also a challenging task because it is an extremely complex and potentially unstable protein, which is capable to aggregate and form multiple oligomeric forms. The results of ELISA assays are strongly influenced by the storage and processing condition of urine samples [38]. Moreover, it was reported that not only the total level of uromodulin in urine is relevant, but the ratio of different oligomeric forms can be significant for early prediction of urolithiasis [8,9], whereas ELISA is not capable of separate determination of the uromodulin oligomers and valuable diagnostic information can be lost. Analytical techniques based on physical separation and detecting of uromodulin molecules in fresh untreated urine can be a potential solution of this problem. Usually DLS is used [8,9,39] for this purpose, but it is a research-grade method, which is almost impossible to implement in a microfluidic device, and alternative approaches have to be suggested. 

Multiple acoustic wave biosensors have been developed for various urinalysis applications, including quantitative determination of urinary globular proteins (microalbumin, α_1_-microglobulin, β_2_-microglobulin, and immunoglobulin G) [40], urea [41], creatinine [42], tetrahydrocannabinol [43], anti-apoptotic protein B-cell lymphoma 2 (Bcl-2) [44], and other biomarkers or trace amounts of pharmaceutical substances [45,46]. Such sensors allow routine determination of low molecular weight metabolic waste products (urea, creatinine) and urinary proteins, which is extremely important for early diagnosis and monitoring of progression of diabetic nephropathy and other forms of chronic kidney dicease (CKD) [40]. Despite high sensitivity and selectivity acoustic sensors for urinalysis are not widely used in clinical practice because other competing, often cheaper, techniques, e.g., test stripes and microfluidic devices with optical detection, are readily available and universally accepted. In this context, it is more justified to create novel acoustic sensors with unique capabilities, which is not achievable in other POC devices, for example, simultaneous determination of as many urine constituents as possible in one stage (albumin and uromodulin, albumin and immunoglobulins, albumin and creatinine etc) or separate determination of physically different but chemically indistinguishable molecules (uromodulin oligomers).

The purpose of this research is the development of the concept and validation of a shear bulk acoustic resonator biosensor with electrical field manipulation for detecting and qualitative determination of abundant urinary proteins with the unique possibility to selectively detect uromodulin oligomeric forms.

In this article, we accented attention to acoustic resonance sensors as one of the most suitable devices for solving the described problem. Acoustic resonant sensor principles are broadly used as biochemical sensors. They are typically understood as a mass sensor [47,48] and therefore called microbalance with the quartz crystal microbalance (QCM) being its most prominent sensor. However, the mass concept hardly describes the sensor when it interfaces liquid and biological medium [49]. According to the work of Kanazawa [50], a liquid defined at the QCM interface exposes an effective load for the resonator due to the finite in-liquid penetration depth of shear evanescent wave. In [51], for the first time, a method for the separate viscosity and density measurements of liquids using QCM was proposed. Shear bulk acoustic resonators are commonly used for biosensing due to low in-liquid radiation losses. This allows keeping the relatively high-quality factor of the resonant sensor even while interfacing attenuating liquids. QCM is a recognized technology that is used to detect interactions at the surface [52]. Excited at a shear bulk vibration mode, its resonant frequency shifts in accordance with interface loading. For bioliquid characterization, QCM was used to detect proteins in some works [53,54,55]. Some modern QCM biosensors use a sensitive or recognition layer to enhance sensitivity and selectivity for specific ingredients in liquid sample. Such QCM biosensors use predefined biointerfaces to detect the proteins [54,56] specifically.

In our study, we focus on an alternative approach that does not require the biointerface, because it is especially challenging to determine the different oligomeric forms of the same protein, e.g., uromodulin, which are chemically identical and could be separated only by physical methods. For the selective detection of different types of proteins, we propose to use a shear bulk acoustic resonator sensor with an additional electrode on the upper part of the bioliquid-filled channel for protein electric field manipulation, which causes modulation of the protein concentration over time in the near-surface region of the acoustic sensor, allowing proteins to be distinguished based on their differences in diffusion coefficients (or sizes) and zeta-potentials. In addition, in order to decouple density contribution from overall sensor response, we propose to use a structured surface of a quartz resonator.

The aim of this work was to show the idea of an alternative biosensor, describe its physical principles of functioning and evaluate its potential. The degree of reliability of the numerical methods and models used is determined by our previous works in development and both theoretical and experimental investigations of microacoustic biosensors and chemical acoustic liquid sensors in which the good agreement of the simulation results with experimental data has been achieved [57,58,59].

The proposed technique and acoustic sensor structure with electric field manipulation have potential applications as a non-disposable POC urinalysis device for early diagnosis and screening of urolithiasis (by the ratio of uromodulin dominant oligomeric forms [8,9,39]) and renal conditions associated with selective proteinuria (by the albumin–immunoglobulins ratio [25,26,27]). It could benefit patients as a new simple, cheap, and easily accessible diagnostic tool, which can be used at a general practitioner’s office or even in home environment.

## 2. Materials and Methods

### 2.1. Properties of Urine and Its Dominant Proteins

Unlike other bodily fluids, e.g., blood serum, whose pH and electrolyte composition are relatively stable due to haemosthasis, physical and chemical properties of urine vary in a wide range depending on diet, fluid intake, physical activity, pathological conditions etc. In healthy individuals urine pH = 4.5–8.0 [60] (typical pH is usually reported as 6.0 [61]); the ionic strength (IS) is 0.07–0.25 [62] (typical IS is close to 0.2 [63]); the normal range for specific gravity (density) is 1.005–1.030 (1005–1030 g/L), typically 1.015–1.025 (1015–1025 g/L) [64]. Urinary infections and many methabolic diseases could lead to abnormally acidic (pH < 4.5) or alcaline (pH > 8.0) urine [60].

Physical characteristics of protein molecules (geometry, morphology, electrical charge, and z-potential, diffusion coefficient) strongly depend on the properties of the surrounding medium, primarily pH and ionic strength of protein solutions. This fact may present a significant challenge for developing POC devices for urinalysis, especially for analysing native urine without additional sample preparation. It should be noted specailly that even for well investigated glomelular proteins such as albumin or immunoglobulins data concerning physical properties at various conditions (pH and IS) can vary considerably in different sources. 

Albumin molecular weight is 66.5 kDa [65,66]; the density is 1334.7 kg/m^3^ [67]; the density of 2 % solution is 1011.8 kg/m^3^ [68] (for this study we assume that the properties of human serum albumin (HSA) and bovine serum albumin (BSA) are very similar [69]). In solutions, albumin is able to adopt different conformations, which are modified by changes in pH or ionic strength; at normal pH the molecule geometry is close to a 9 × 5.5 × 5.5 nm spheroid [70], but the length of the molecule changes from 7 to 14 nm depending on the pH [71]. The hydrodynamic radius of albumin in water solutions is 4.3 nm [72], but at high concentrations it reduces [73]; some authors report values as low as 2.7 nm, which can be attributed to possible dimerization [67]. The diffusion coefficient of albumin in water solution was estimated as 6.1 × 10^−7^ cm^2^/s (pH = 6.3) [74]. The isoelectric point of an albumin molecule is pI = 5.0, the z-potential varies from −50 to 50 mV depending on the pH and IS [73] (for higher IS characteristic for urine smaller values from 12 to −20 mV are observed [75]). 

Immunoglobulins G have a higher molecular weight of 156 kDa and hydrodinamic radius of 5.5 nm [27] than albumin, the molecules are Y-shaped and 10–15 nm long [76]. The isoelectric point of IgG is pI = 7.0–9.5 (with the exception of the IgG4 fraction, which constitutes only 4% of all IgG in serum and urine) [27]; the absolute values of IgG zeta-potential is close to that of albumin [77]. As expected, the diffusion coefficient of globulins 3.89 × 10^−7^ cm^2^/s (non-fractioned IgG, pH = 7.4 [78]) is lower than the diffusion coefficient of albumin.

Uromodulin oligomeric form with 7 MDa molecular weight (lighter fraction) takes form of a thread-like macromolecule, which is 600 nm long and 5 nm in diameter; in solution, it gathers into a ball with a hydrodynamic radius of 100 nm. The heavier fraction with a molecular weight of 28 MDa takes form of a 1200 nm long rod, 8–10 nm in diameter, it has a rigid structure and does not form a coil; the hydrodynamic radius of this oligomer is about 400 nm [8,39,79,80].

The 7 MDa molecule has a z-potential of −30–26 mV, the z-potential of the 28 MDa molecule is significantly lower: From −8 to −2 mV (pH = 5.5–6.5) [39]. The isoelectric point for both fractions is below the pH of most acidic urine pI < 3 [62], the diffusion coefficient is much smaller compared to globular proteins—0.22 × 10^−7^ cm^2^/s (pH = 8) [81].

Along with the basic molecular properties in solutions, spontaneous adsorption of proteins onto liquid–solid interfaces should always be considered thoroughly while developing various sensors for analysis of biological fluids because it strongly influences chemical, mechanical, and electrical properties of metal and dielectric surfaces. This phenomenon has been extensively investigated both theoretically and experimentally [82,83,84]; a plethora of biochemical, radiochemical, electrochemical, optical, and acoustic techniques have been applied including enzyme-linked immunosorbent assay (ELISA) [85], radiolabelling [86], ellipsometry [87], total internal reflection fluorescence (TIRF) [88], surface plasmon resonance (SPR) [89], electrochemical quartz crystal nanobalance (EQCN) [90], and quartz crystal microbalance (QCM) [55,91], and polarization modulation infrared reflection absorption spectroscopy (PM-IRRAS) [92].

The rate and the other characteristics of adsorption and consequent desorption processes depend on many factors, including the pH, the IS, the properties of the protein molecules and the interfaces, the nature of the solvent and other components present. Protein molecules are polypeptide chains with complex three-dimensional spatial structure, which could be a coil, α-helix, β-pleated sheet, or sphere (globular proteins). Side chains of different amino acids in a molecule could be hydrophilic and hydrophobic, making proteins amphiphilic, capable to adsorb both unto hydrophilic and hydrophilic surfaces. It is almost impossible to develop a common theory of protein adsorption, usually adsorption of a specific group of proteins (e.g., globular proteins) onto a specific type of surfaces (e.g., metals or quartz) [93].

Uromodulin has helix and coil secondary structure [94,95]; albumin and immunoglobulins are globular plasma proteins [96]. It should be expected that uromodulin and globular proteins are characterized by different adsorption behavior, but while adsorption properties of globular proteins, especially albumin, are well investigated [82,83,84], the surface activity of uromodulin is still relatively poorly understood.

As any other biological fluid, urine is an extremely complex medium, containing thousands of various components—low molecular weight metabolic products, electrolytes, macromolecules (including proteins), biological cells, urinary casts, etc. [97]; mathematical simulation of physical processes in such media is an almost unrealizable task, especially at a micro level. Because of this fact at the initial stage of the development of an analytical device for urinalysis it is reasonable to analyze a simplifed model solution with the pH and IS close to that of native urine. In this research water solution of albumin, immunoglobulin G and uromodulin oligomeric forms at relevant concentrations are considered; sodium chloride [98] and bicarbonate ions [99] are also introduced to the medium to achieve the necessary IS and pH, respectively.

### 2.2. Sensor Structure

Taking into account the complex composition of urine and the significant variability of its characteristics (pH, electrolyte composition, different types of proteins, low molecular weight of content), it is advantageous to apply a multiphysics approach that com-bines interaction in electrical and acoustic fields. The following requirements are imposed on the device and method being developed:

- High-quality factor of the resonant sensor;
- the microfluidic device material must be bio-competitive and possibly reusable; and
- the design, manufacturing method, and operating principle must be robust so that the device can be used for routine measurements.

**Figure 1:**
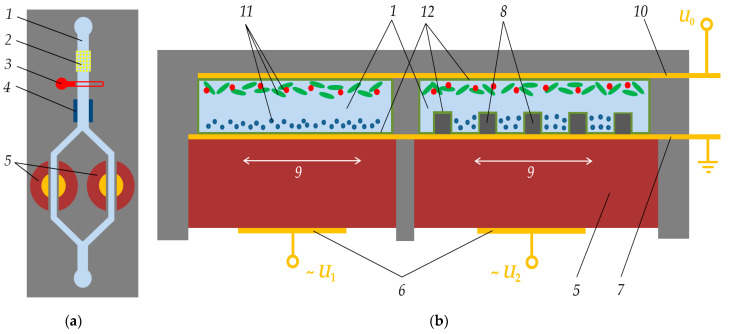
Top (**a**) and slice (**b**) view of a schematic model of the device for manipulating and detecting proteins: *1*—Biological liquid (urine) in the canal; *2*—filter; *3*—temperature control; *4*—*pH* control; *5*—piezoelectric quartz crystal resonators; *6*—bottom electrodes under alternating probing potential *U*_1_ and *U*_2_; 7—top grounded electrode; *8*—set of protrusions placed on the top electrode surface; *9*—direction of shear vibrations of a quartz resonator, perpendicular to the microfluidic channels and a set of top electrode protrusions; *10*—addition electrode over the channel for particles manipulations in liquid due to the application of electric potential *U*_0_; *11*—different types of protein particles; and *12*—surface passivation layer. Geometrical proportions of the figures are not observed for reasons of visual clarity (e.g. quartz crystal must be much thicker; many tens of periods of protrusions must fit on the area of the top electrode; particles must be smaller in reality).

The schematic representation of the proposed microfluidic device is shown in Figure 1. In a channel filled with a biological fluid, there are two shear bulk resonant sensors, one with a planar surface and the other is featured with a structured surface. An additional electrode is located above the resonators, to which the potential can be applied. By changing the electric potential of this additional electrode, protein concentration near the surface of the sensors is modulated according to their sizes and zeta-potentials. Depending on the sensor surface structuring, the sensor response will be affected by different loads and varying degrees of sensitivity to the properties of the liquid and proteins in it (density and viscosity). This opens up wide opportunities for tuning the sensor device for a specific object under study.

### 2.3. Numerical Study Methodology

**Figure 2:**
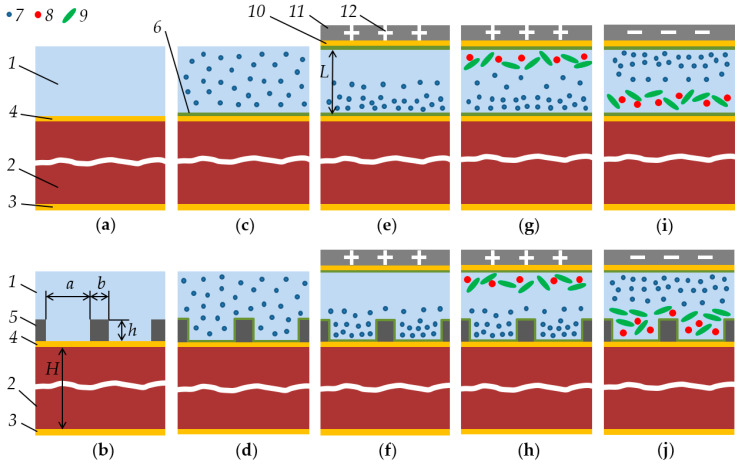
Model development of quartz resonator with planar (**a**,**c**,**e**,**g**,**i**) and structured (**b**,**d**,**f**,**h**,**j**) top surfaces with direct solid/liquid contact (**a**,**b**) or coated with a thin surface passivation layer (**c**–**j**) and loaded by liquid in different cases: (**a**,**b**) Homogeneous liquid; (**c**,**d**) liquid with protein particles of one type that are evenly distributed throughout the liquid volume; (**e**,**f**) liquid with charged particles of one type that are concentrated near the quartz surface due to an external electric field; (**g**,**h**) liquid with several types of particles separated in external electric field made by positively charged additional top electrode, which concentrate positively charged particles near the quartz surface and sorts out negatively charged particles; (**i**,**j**) liquid with several types of particles separated in external electric field made by negatively charged additional top electrode, which concentrates negatively charged particles near the quartz surface and sorts out positively charged particles. Schematic model of sensor structure consists of measured liquid (1); quartz crystal resonator (2) of *H* thickness; bottom electrode (3) under alternating probing potential; top grounded electrode (4); set of protrusions (5) of *h* height, *a* distance and *b* width, placed on the top electrode surface; surface passivation layer (6); positively (7) and negatively (8, 9) charged protein particles of different mass and morphology; addition electrode (10) attached to the *L* height ceiling of the microfluidic channel (11) and charged (12). Geometrical proportions of the figures are not observed for reasons of visual clarity (e.g. particles must be smaller in reality).

The sequence of the numerical analysis performed in this work is shown in Figure 2 and consisted of several methodological steps dedicated to the study of shear bulk acoustic resonators with planar and structured surfaces in contact with liquid that is:

- Homogeneous;
- containing evenly distributed protein particles; and
- containing protein particles under the influence of the external electric field.

Numerical simulation was performed in COMSOL Multiphysics software using the coupled physics to study piezoelectric solid–fluid complex interaction in an applied electrical field.

## 3. Theory

The sensor is designed as a shear bulk acoustic resonator made of AT-cut quartz crystal plate. Two thin metal electrodes are defined on both sides of the quartz crystal plate. Thickness-shear vibrations are produced by applying a periodic potential to the electrodes. Its resonance frequency ($f_{q}$) depends on the shear-wave velocity ($v_{s}$) and the quartz thickness (*H*). The frequency of fundamental vibration mode can be calculated as follows [47]:

(1) $$f_{q}=v_{s}/\left(2H\right).$$

Sauerbrey et al. [48] have described the frequency shift (Δ*f*) of the fundamental resonant frequency of quartz resonator when it is loaded by a small additional mass (Δ*m*) per area of the electrode (*A*) according to the equation:

(2) $$\Delta f_{m}=-\frac{2f_{q}{}^{2}}{\sqrt{\rho_{q}\mu_{q}}}\frac{\Delta m}{A},$$

where $\rho_{q}$ is the density of quartz; $\mu_{q}$ is the elastic constant of the piezoelectrically stiffened quartz.

Small mass and high Q-factor of the resonator give access to a mass detection limit Δ*m*/*m*_res_ of 10^−6^ to 10^−9^ with Δ*m* and *m*_res_ being the added mass and resonator mass per unit area, respectively.

When a quartz crystal sensor is in direct contact with a liquid, the shear wave penetrates into it to a small depth and the frequency shift is determined by the acoustic properties of the near-surface liquid with the thickness of δ = (η/πρ*f*)^1/2^, where η and ρ are the liquid viscosity and density. According to the work of Kanazawa [4], a liquid defined at the QCM interface exposes an effective load for the resonator due to finite in-liquid penetration depth of shear evanescent wave:

(3) $$\Delta f_{L}=-\frac{f_{q}{}^{3/2}\sqrt{\rho \eta }}{\sqrt{\pi \rho_{q}\mu_{q}}},.$$

For reliable separate measurements of liquid sample viscosity and density, in [51], a method for comparing the results of measuring a liquid using smooth and corrugated electrodes of QCM was proposed. The respective frequency shifts for a smooth- (Δ*f*_L1_) and corrugated-surface (Δ*f*_L2_) QCM liquid sensors are given as [51]:

(4) $$\Delta f_{L1}=-c_{1}\sqrt{\rho \eta };\;\Delta f_{L2}=-c_{1}\sqrt{\rho \eta }-c_{2}h\rho ,$$

where *c*_1_ and *c*_2_ are constants, *h* is the depth of the grooves on the corrugated surface of QCM electrode. The constant *c*_1_ can be calculated from Equation (3). The constant *c*_2_ depends on material properties of surface structural elements, geometric parameters and can be determined experimentally or numerically. Equation (4) corresponds to the cases in Figure 2a,b. The difference between the shifts of the resonant frequencies of these two devices makes it possible to determine the average density of the liquid trapped between the elements of the structured surface of *h* height:

(5) $$\Delta f_{D}=\Delta f_{L1}-\Delta f_{L2}=c_{2}h\rho .$$

To determine the effect of the protein content in a biological liquid on the shift of quartz resonators (cases in Figure 2c,d), it is necessary to determine the density and viscosity as a function of the protein concentration. One of the ways to calculate these values is as follows [100,101,102,103]:

(6) $$\rho =\rho_{0}\left(1-x_{p}\right)+\rho_{p}x_{p};\;\eta =\eta_{0}{\left(1-\frac{x_{p}}{x_{\mathrm{pm}}}\right)}^{-\left[\eta \right]x_{\mathrm{pm}}},$$

where ρ_0_ and ρ_p_ are the liquid medium and protein molecules densities; *x*_p_ and *x*_pm_ are the volume fraction of the protein molecules and its maximum value; η_0_ is the liquid viscosity without proteins; [η] is the constant depending on the protein type.

The electrical field proteins manipulation corresponds to the constant potential mode, and the separation efficiency depends on the values of the zeta-potential and diffusion coefficient (which is inversely proportional to the hydrodynamic radius of protein particles), as a result, the electrophoretic mobility of proteins, could be determined from Smoluhovsky equation [104,105,106]:

(7) $$\frac{\partial }{\partial t}G\left(r;r_{0};t\right)=\nabla \cdot \frac{1}{m\xi }G\left(r;r_{0};t\right)\nabla \varphi \left(r\right)+\nabla \cdot D\nabla G\left(r;r_{0};t\right),$$

where $G\left(r;r_{0};t\right)$ is particle distribution function of coordinates and time; ∇ is nabla operator; φ(r) is potential distribution; *D* is the diffusion constant and *ξ* is the friction coefficient on the Smoluchowski timescale. Despite the seeming simplicity of this approach, it is necessary to take into account that the values of the zeta potential depend on many factors (first of all, the pH of the medium and the concentration of electrolytes), which in native urine can vary widely both in normal conditions and in pathology. In particular, the urine pH of a healthy person can range from 4.5 to 8.0. In addition, the Smoluchowski equation, which uniquely links electrophoretic mobility with zeta potential, was obtained for spherical particles, which is incorrect for the proteins, so in the case under consideration it gives only approximate results. These factors lead to the need for extensive research to determine the electrophoretic mobility of proteins under conditions of different environmental parameters.

The solution of Equation (7) allows finding the distribution of proteins near the surface of the quartz resonator. In this case, the parameters of the liquid (density and viscosity) become a function of coordinates and time (cases in Figure 2e,f). In [107], a model was proposed for calculating the response of a quartz resonator under the action of a multilayer viscoelastic load, when the viscosity and density of quartz coatings change from one layer to another. However, even in this simplified representation, the behavior of a quartz sensor under an anhomogeneous load can only be determined by numerical calculations. The presence of various types of protein molecules (cases in Figure 2g–j) requires the solution of a system of equations for each particle, taking into account their electrochemical interaction. In the present work, this was done using numerical simulations in COMSOL Multiphysics Software.

In general, the resonant frequency (*f*_r_) of a quartz crystal sensor will be the sum of the following components:

(8) $$f_{r}=f_{q}+\Delta f_{m}+\Delta f_{\mathrm{pl}}+\Delta f_{L}+\Delta f_{D},$$

where $\Delta f_{\mathrm{pl}}$ is the frequency shift due to passivation coating. It can be determined by subtracting the resonance frequencies of the sensor structure measured in distilled water without and with a passivation coating. But the most important and informative is the $\Delta f_{D}$ shift, which is easily determined by measuring the differential response from quartz resonators with smooth and structured surfaces, which provides the key to quantifying the concentration of proteins in a biological liquid.

## 4. Results of Numerical Modelling

### 4.1. Modelling the Response of a Sensor to a Change in the Properties of a Homogeneous Liquid under Various Conditions of the Surface Structuring

**Figure 3:**
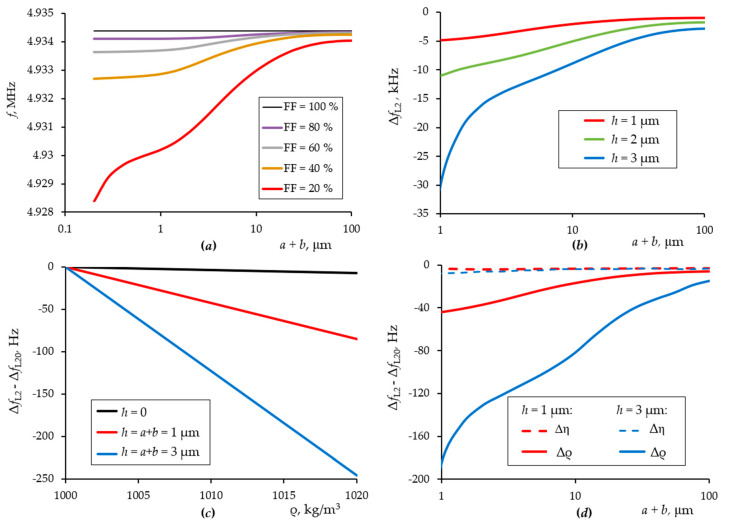
Resonant frequency of a quartz crystal sensor depending on the method of structuring its surface at various filling factors and an average height of 200 nm (**a**); shift of the resonant frequency of a quartz resonator, depending on the method of structuring its surface at a filling factor of 20 % and different block heights (**b**); resonator frequency shift depending on liquid density for different heights of structural elements (**c**); frequency shift of a quartz resonator with structured surface elements of 1 and 3 µm height with a change in the liquid density and viscosity of 1 % (**d**). Subscript “0” means original liquid parameters.

At the first stage, a homogeneous liquid solution with properties close to distilled water was considered. The influence of the method of structuring the surface of a quartz resonator on the sensitivity to the density and viscosity of the liquid was investigated (Figure 3). The geometric parameters of the structured surface layer were: *a* is distance between blocks; *b* is block width; *h* is block height; FF = *b*/(*a* + *b*) is filling factor of the electrode surface with structural elements; *h*_av_ = *h*·FF is the average height of the structured layer.

Figure 3a shows simulation results for the change in the resonant frequency of a quartz crystal with a change in the surface structure period and filling factor (corresponding to Figure 2b case). The average height was kept constant, which ensured the constancy of the mass load on the quartz. The average height was taken small, only 200 nm, which should ensure the operation of quartz in a linear mode, according to classical concepts. Despite the constancy of the total mass of the layer, the method of structuring the surface layer significantly shifts the resonance frequency. An increase in the height of the structure enhances this effect (Figure 3b). Figure 3c shows how significantly the sensitivity of the sensor to the density of the liquid can be changed by manipulating the surface structure. At the same time, the sensitivity to viscosity remains practically unchanged (Figure 3d).

The calculation results show that with an increase in the height of the structural elements and a decrease in their width, it is possible to significantly increase the sensitivity of the sensor to the density of the liquid trapped between the structural elements, while the sensitivity to the viscosity of the liquid remains constant. That makes it possible to create an acoustic sensor that is sensitive to the changes in the mass density of particles dissolved in a liquid. The limitation is the technological possibilities of creating a structured quartz surface with very narrow and high structural elements so that they can move synchronously with the vibrations of the quartz surface without significant bending since bending will lead to additional acoustic losses, which will neutralize the effect of hypersensitivity to liquid density. In Figure 3a,b, this is clearly seen at low values of (*a*+*b*), when the dependence acquires a large slope. This means that a further decrease in the structure period value is unjustified. In particular, for this reason, it is preferable to use rigid materials with low acoustic losses.

The shift of the resonance frequency of the quartz plates is in agreement with Equation (5); however, the coefficient *c*_2_ is a function of the height and width of the structural elements. For further modelling, we chose the height and the filling factor of structural elements of 3 μm and 20 %, respectively. It was assumed that the structural elements are made of glass. The operating frequency range of the quartz sensor structures for the proposed model is from 4.86 to 4.88 MHz, since a fundamental frequency of pure quartz resonator is about 5 MHz.

### 4.2. Modelling of the Separation and Detection of Albumin and Globulin

**Figure 4:**
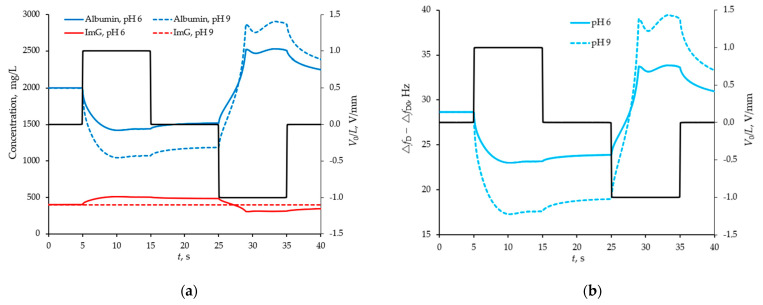
Average concentration of proteins (shown by red and blue curves) trapped in the space between the barriers of the sensor with the structured surface at two different *pH* values (**a**) and the difference response from two sensors (with smooth and structured surfaces) in the form of a shift (shown by blue curves) of their resonance frequencies (**b**) by manipulation of the model buffer solution with proteins by external pulsed field (indicated by a black broken line).

**Figure 5:**
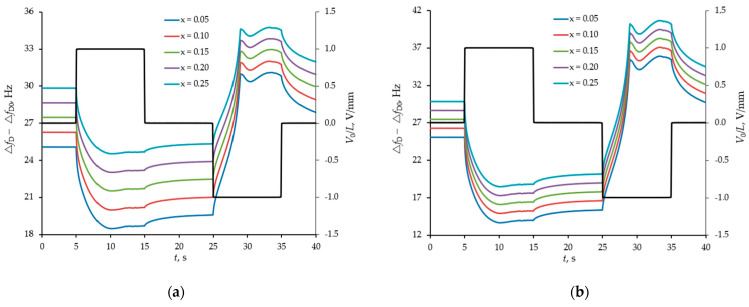
The difference response from two sensors (with smooth and structured surfaces) in the form of a shift of their resonance frequencies to manipulation of a model buffer solution with proteins by an external pulsed field at different content of globulin (x = [ImG]/[Albumin], [Albumin] = 2 g/L) for pH = 6 (**a**) and pH = 9 (**b**). Frequency shifts (left axes) are shown with coloured curves, and pulse actions (right axes) are shown with a black broken line.

The following model protein solution with a controlled pH level was considered. The parameters of water at 20 degrees Celsius were taken as the density and viscosity of the medium. The pH of the medium was taken into account on the basis of the water dissociation reaction and was controlled by bicarbonate ions concentration at a level from 6 to 9. The ionic strength of the solution was taken into account by introducing sodium and chlorine ions with a total concentration of 60 kmol/L. The albumin concentration was taken at the level of 2 g/L, and the globulin level varied from 5 to 25% relative to the albumin concentration. The compositions of the model solution corresponded to the cases of selective and non-selective macroproteinuria. The temporal dynamic response of the sensor to the modulation of the protein concentration near the sensor surface was calculated when the solution was exposed to oppositely polarized pulses of an electric field. The simulation results are presented in Figure 4 and Figure 5.

Figure 4 and Figure 5 show that the efficiency of selective detection of albumin and globulin depends on the pH of the medium. So, for example, at pH = 6, albumin and globulin have different signs of the zeta-potential, about −12 and +12 mV, respectively, while at pH = 9, globulin is in the vicinity of the isoelectric point and does not lend itself to the influence of an electric field. For this reason, with a decrease in the pH of the model solution, the efficiency of selective detection of albumin and globulin is higher.

### 4.3. Modelling of the Separation and Detection of Oligomeric Forms of Uromodulin

**Figure 6:**
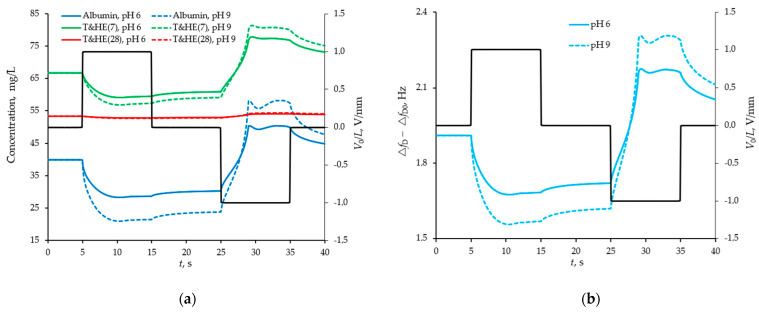
Average concentration of proteins trapped in the space between the barriers of the sensor with the structured surface at two different pH values (**a**) and the difference response from two sensors (with smooth and structured surfaces) in the form of a shift of their resonance frequencies (**b**) by manipulation of the model buffer solution with proteins by external pulsed field. Concentrations and frequency shifts (left axes) are shown with coloured curves, and pulse actions (right axes) are shown with a black broken line.

Further, a more complex case of the separation of oligomeric forms of uromodulin was considered taking into account different background levels of albumin. The pH and ionic strength of the model solution was controlled in the same way as in the previous case. The separation of oligomeric forms of uromodulin is possible due to their significant difference in sizes, and therefore in the magnitude of the hydrodynamic radius and diffusion coefficients, respectively. A heavier protein turns out to be much less mobile in an external electric field (see Figure 6a).

**Figure 7:**
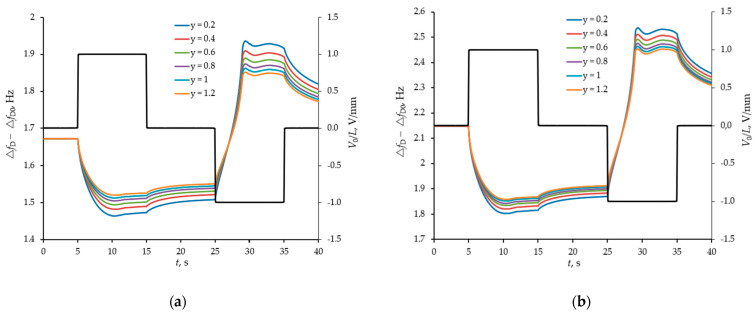
The difference response from two sensors (with smooth and structured surfaces) in the form of a shift of their resonance frequencies to manipulation of a model buffer solution with proteins by an external pulsed field at different ratios of oligomeric forms of uromodulin (y = [T&HE (28)]/[T&HE ( 7)], [T&HE (28)] + [T&HE (7)] = 120 mg/L) for a normal albumin level of 20 mg/L (**a**) and for its 3-fold increase (**b**). Frequency shifts (left axes) are shown with coloured curves, and pulse actions (right axes) are shown with a black broken line.

The pH level also affects the efficiency of selective detection, but not significantly in the range of pH from 6 to 9, since all proteins considered in the model (albumin and two oligomeric forms of uromodulin) have the same zeta-potential signs. Background albumin is more important. Figure 7 shows the temporal responses of the sensor at normal albumin levels and a threefold increase. The higher the background level of albumin, the lower the efficiency of selective detection of oligomeric forms of uromodulin.

### 4.4. Recommendations for the Application of the Approach for Protein Analysis

Based on the analysis carried out, the following method of working with a sensor device can be proposed.

At the first stage, the sensor device must be filled with distilled water and measured its frequency characteristics. Then add a solution for deposition a passivating coating. After some exposure, the solution should be removed and again filled with distilled water and repeated measurements. By comparing the frequency shifts of the first two measurements, the effect of the passivation coating on the frequency shift can be subtracted. Next, a biological fluid solution is introduced, and its frequency characteristics are measured under the pulsed action of an external electric field. The difference shift of the resonance frequencies from two acoustic resonator sensors, according to equation (5), makes it possible to estimate the average density of the liquid with proteins trapped between the barriers of the structured surface of the quartz resonator. From that mass density, it is possible to calculate the concentrations of protein particles in the solution by comparing the sensor response with the simulation results. Thus, the success of this approach is associated with the development of fairly accurate models of the behavior of proteins in biological fluids.

## 5. Discussion

As a basis and starting point of the current study, the QCM technique was chosen because it is a recognized biosensor technology that is used to detect specific interactions at the sensor surface when the sensor is featured with a specific biointerface that binds ligands of interest from interfacing medium. The biochemical reaction is mostly irreversible with a detected components remained at the biointerface. Our study focuses on alternative approach that does not require binding of biomolecules to predefined biointerface.

In our sensor concept, we apply the near-field micro-acoustic sensing principle. In comparison to far-field sensors that rely on propagating wave, the near-field sensor operates at non-radiating conditions with elastic vibrations confined within the resonant sensor structure. The measured resonant frequency of the sensor depends on the acoustic loading imposed at the interface near the sensor surface, such as overlayer or/and interfacing liquid. To confine the elastic vibrations, we excite the sensor structure at shear bulk vibration mode. Liquids do not support the propagation of shear wave that enables efficient confinement of the elastic vibration within the sensor structure that interfaces liquid medium. A liquid defined at the QCM interface exposes an effective load for the resonator due to the finite in-liquid penetration depth of shear evanescent wave. Using the structured surface of the sensor, we not only get the opportunity to separately measure density and viscosity (as shown in [51,108,109,110]), but also allow the acoustic field to penetrate deeper into the liquid (to the height of structural elements), thereby increasing the sensor’s sensitivity to small changes in liquid density (which is possible with the certain choice of height, filling factor, and material of structural elements, as shown in 4.1). We propose to couple shear bulk acoustic resonator type of sensor with protein electric field manipulation, which causes modulation of the protein concentration over time in the near-surface region. Numerical simulations showed that the proposed approach allow distinguish proteins in solution based on their differences in diffusion coefficients (or sizes) and zeta-potentials.

In the current work, it was shown by computer simulation, that the combination of electrical field manipulation and acoustic resonance detection in the framework of a POC device without any biointerface allows reliable determination of the concentration ratio of uromodulin oligomeric forms y = [T&HE (28)] / [T&HE (7)] (macromolecules with molecular masses 7 MDa and 28 MDa) and the IgG/albumin ratio in model solutions imitating urine at physiological conditions (pH = 6.0–9.0, typical urine ionic strengh, normal or slightly elevated albumin level, normal or slightly elevated total uromodulin level). It can be concluded from the simulation results that the proposed technique and sensor structure make it possible to quantitatively determine the ratio of uromodulin oligomers in the range y = 0.2–1.2; higher values indicate onset and progression of urolithiasis, even when no specific symptoms are manifested [9]. A higher IgG/albumin ratio can indicate increased kidney damage for diabetic patients with CKD [26,27]. As an obvious limitation of the techinique, it should be noted, that the sensitivity of the sensor structure to the concentration ratio of uromodulin oligomeric forms decreases in case of significant albuminuria.

In this numerical study, we have shown the typical characteristics of the sensor structure that can be achieved by standard technological methods. The sensitivity of the sensor can be increased by optimizing the geometry and choosing the materials of the structured layer, as well as by performing measurements in a nonlinear mode, on overtones and using special mathematical processing of frequency spectra.

The most science-intensive and innovative part for the future research is the use of structuring effects, as well as taking into account various types, their shapes, sizes, mass, and surface potential of proteins and pH of the medium for analysing the results.

## 6. Conclusions

The article presents the idea and physical principles of alternative biosensor concept. In this contribution, we have proposed the sensor approach that combines acoustic and electrical methods for label-free separation and detection of proteins for potential application in routine urinalysis diagnostics. The method is based on near the surface detection with shear bulk acoustic resonator approach with a structured surface for enhanced mass density sensing. The applied electrical field allows to modulate the surface concentration of targeting protein and detect its concentration. This method was described, recommendations were made on its use, sensor characteristics and perspectives were estimated, sensor responses were calculated using the example of urine models.

The results of numerical studies show the following:

- Structuring the surface of a quartz resonator based on the certain choice of height, filling factor and material properties of structural elements can significantly increase the sensitivity of the shear acoustic near-field detection method to small changes in liquid density;
- the most informative are the measurements of the difference between the resonant frequencies of quartz resonators with smooth and structured surfaces as a function of time under a multipolar impulse action on the bioliquid analyte with controlled pH;
- the ability of the proposed sensor concept to separate and specifically detect oligomeric forms of uromodulin, albumin and globulin;
- the proposed approach allows to quantitatively determine the ratio of uromodulin oligomers, however the sensitivity of the sensor structure to the concentration ratio of uromodulin oligomeric forms decreases in case of significant albuminuria;
- interaction in acoustic and electric fields allows to amplify the sensor response to the presence of targeting protein, but the values of expected frequency shift vividly show the requirement of high-quality factor resonant biosensor performance; and
- in addition to solving design requirements, the success of the proposed approach is associated with the development of sufficiently accurate models of the behaviour of proteins in biological fluids in electric field with the aim to be able to correctly interpret sensor time dependent frequency characteristics and extract reliable information on their basis.

A non-disposable POC urinalysis device for early screening and monitoring of progression of urolithiasis could be developed using the proposed acoustic sensor structure, with obvious benefits for patients and general practitioners as a simple, inexpensive, and easy-to-use diagnostic tool.

In the upcoming work, the experimental studies will be performed to validate the proposed sensor concept.
